# Solute Transport across the Lymphatic Vasculature in a Soft Skin Tissue

**DOI:** 10.3390/biology12070942

**Published:** 2023-06-30

**Authors:** Dingding Han, Ziyang Huang, Ehsan Rahimi, Arezoo M. Ardekani

**Affiliations:** 1 School of Mechanical Engineering, Purdue University, 585 Purdue Mall, West Lafayette, IN 47907, USA; han608@purdue.edu (D.H.);; 2 Mechanical Engineering Department, University of Michigan, Ann Arbor, MI 48109, USA

**Keywords:** solute transport, lymphatic vasculature, improved Kedem–Katchalsky model, poroelasticity, computational fluid dynamics

## Abstract

**Simple Summary:**

The lymphatic system plays a crucial role in maintaining fluid and solute balance in biological tissue, which makes the subcutaneous injection a common approach for delivering therapeutic agents through the lymphatic pathway. The transport of drug solutes is regulated by the interstitial fluid pressure. In our study, a time-efficient poroelastic model is developed to mimic pressure relaxation and build-up in the soft skin tissue. This method can accurately and time-efficiently capture the evolution of pressure, which is validated against both the analytical solution and numerical solution of the previous poroelastic model. The increasing porosity and permeability due to deformation alleviate the high pressure caused by the injection. Furthermore, an improved solute transport model is developed to better address the microscopic properties of the lymphatic vessel membrane. The effects of a varying Stokes radius of drug solute and vessel network structures are investigated on lymphatic uptake. Our comprehensive computation model provides a time-efficient and reliable research tool for studying solute transport into the lymphatic system, which can be utilized to support decision-making regarding lymphatic disturbed diseases.

**Abstract:**

Convective transport of drug solutes in biological tissues is regulated by the interstitial fluid pressure, which plays a crucial role in drug absorption into the lymphatic system through the subcutaneous (SC) injection. In this paper, an approximate continuum poroelasticity model is developed to simulate the pressure evolution in the soft porous tissue during an SC injection. This poroelastic model mimics the deformation of the tissue by introducing the time variation of the interstitial fluid pressure. The advantage of this method lies in its computational time efficiency and simplicity, and it can accurately model the relaxation of pressure. The interstitial fluid pressure obtained using the proposed model is validated against both the analytical and the numerical solution of the poroelastic tissue model. The decreasing elasticity elongates the relaxation time of pressure, and the sensitivity of pressure relaxation to elasticity decreases with the hydraulic permeability, while the increasing porosity and permeability due to deformation alleviate the high pressure. An improved Kedem–Katchalsky model is developed to study solute transport across the lymphatic vessel network, including convection and diffusion in the multi-layered poroelastic tissue with a hybrid discrete-continuum vessel network embedded inside. At last, the effect of different structures of the lymphatic vessel network, such as fractal trees and Voronoi structure, on the lymphatic uptake is investigated. In this paper, we provide a novel and time-efficient computational model for solute transport across the lymphatic vasculature connecting the microscopic properties of the lymphatic vessel membrane to the macroscopic drug absorption.

## 1. Introduction

The lymphatic vascular system returns the interstitial fluid to the systemic circulation in the human body, which plays an essential role in homeostasis and immunization in the human body [1,2,3,4]. Changes or defects in the lymphatic vascular function can induce variable human body responses to a wide range of human diseases, such as obesity and Alzheimer’s disease [4]. The lymphatic pathway is a common approach for the delivery of large therapeutic proteins, such as monoclonal antibodies, through subcutaneous (SC) injection [2,3,5,6]. SC injection has many merits, such as lower cost and convenience compared to intravascular (IV) injection [7,8,9]. In a recent administration analysis, subcutaneous injection takes up 76% of all the US FDA-approved administration of therapeutic agents of high concentration [10]. After being injected into the subcutaneous tissue, drug solutes transport into the initial lymphatics, deeper collecting lymphatics and then lymph nodes, and at last, into the systemic circulation [2]. During the transport process, many internal and external factors affect drug absorption after subcutaneous injection, such as the physiological properties of the subcutaneous tissues, injection site, drug and formulation properties, and administration mode [6]. The quantitative investigation on the subcutaneous absorption through physics-based computational modeling bridges the gap between the understanding of the absorption process through SC injection and the development of drug and drug delivery system.

In our previous works [11,12,13], the convective transport of drug solutes is driven by the interstitial fluid pressure (IFP) into the lymphatic system, which plays an important role in drug absorption through SC injection. The mechanical process of the injection and its effect on pressure build-up and relaxation are essential to understand the fluid flow and drug clearance at the injection site [12,14]. The diffusion across the discrete heterogeneous lymphatic vessel network significantly affects the lymphatic uptake [13]. However, the computation cost is expensive for a multi-scale problem with both a heterogeneous explicit vessel network and the constitutive equations included, where the vessels of radius *O* (10 μm) are embedded in a domain of size *O* (1 cm). A time-efficient and accurate biomechanical model for pressure evolution can be valuable to such a complex computational system to study the transport and absorption of large drug molecules in biological tissues.

The pressure build-up in subcutaneous tissue during the subcutaneous injection has been investigated through experiments in several previous studies [15,16]. The counter pressure in the subcutaneous tissue is estimated based on experimental data, and a model for the pressure evolution is constructed [15]. The experiments of subcutaneous injection show that controlling the injection rate could be used to partially prevent back pressure from increasing to unacceptable ranges, while changing the injection volume alone shows little influence on pressure evolution [17]. The X-ray imaging technique is used to study the effects of injection conditions on the permeation of the drug in tissues [18] in horizontal and vertical directions for different injection speeds. Due to the lack of in situ quantitative experiments on IFP and lymphatic uptake at the injection site, numerical simulations are used to improve our understanding of the effects of the tissue mechanical properties on drug delivery. The effect of the biomechanical responses of the soft tissue on the fluid flow is investigated by coupling Darcy’s law with stress–strain behaviors for the solid skeleton, i.e., the constitutive models. The poroelastic model is widely used to describe the deformation of biological tissues and hydrogels [12,14,19,20]. Our previous studies numerically investigate the contribution of large interstitial pressure to drug absorption during and after the injection [11,12]. A hybrid discrete-continuum vessel network model is developed to describe the diffusion and convection of drug solutes across the vessel wall [13]. Although the poroelastic model can simulate the deformation of the soft subcutaneous tissue, the computation is expensive after coupling with transport equations and instability issues may occur [12]. Including a three-dimensional (3D) heterogeneous vessel network in the simulations can make the computations more expensive. A few methods are used to simplify the computations in the literature. One approach is reducing the dimension of the numerical simulation. The mathematical equations for the interstitial fluid flow and soft tissue strain are solved in two-dimensional models using a finite element method to study the effect of strain relaxation on fluid drainage and solute transport [21,22], while one-dimensional mathematical models are also used to analyze the fluid flow and solute transport [23]. Another approach is simplifying the stress–strain relation to make the computations more efficient. The linear poroelasticity based on small strain theory is often used to simplify the constitutive Equation [24]. In [25], the deformation and strain are obtained by simplifying the poroelastic constitutive equation to simulate the deformation of a hydrogel. In this paper, we develop a new approximate poroelastic model to mimic the relaxation of the pressure in soft tissues.

As one of the two vascular circulatory systems in the human body, the lymphatic vessel network consists of initial lymphatics with only one single layer of lymphatic endothelial cells (LECs), unlike blood vessels which have a complete basement membrane [2,3,4]. For lymphatic vessels, LECs are loosely connected and have a primary valve structure to ensure the one-directional fluid flows from the interstitial space into the lymphatics. In our previous work [13], three different transport conditions are used to investigate solute transport across the lymphatic vessel membrane, including the Kedem-Katchalsky model. In [26,27], an integral form of the modified Kedem–Katchalsky formulation is used to study dermal clearance through the blood and lymphatic vessels. To further address the special physiological structure of the lymphatic vessel membrane, in this paper, we develop an improved Kedem–Katchalsky model for solute transport across the explicit lymphatic vessel wall. The morphology of the initial lymphatic vessel network varies over the location and species [3]. In human skin tissue, blind-ended and interconnected structures (reticular plexus) are observed for initial lymphatic vessels [28,29,30,31]. However, there is no quantitative investigation on the effect of various structures of the lymphatic vessel network on solute transport and absorption through the lymphatic vessel network.

In this paper, an approximate continuum poroelastic model is developed to simulate the pressure evolution in the soft subcutaneous tissue. The pressure evolution of the proposed model is quantitatively validated against the analytical solution and the numerical solution of the poroelasticity model. The effect of elasticity, variable porosity and permeability on pressure build-up is numerically investigated. The convective and diffusive transport of drug solute into the lymphatic vessels is investigated through an improved Kedem–Katchalsky model by implementing the hybrid vessel network in the continuum poroelastic model. At last, heterogeneous vessel networks with various structures are used to investigate the effect of the morphology of the lymphatics on solute transport into the lymphatic system in the multi-layer soft skin tissue.

## 2. Methodology

To simulate the microcirculation in the subcutaneous tissue, the physics-based computational model established in this study is combined with physiological models, i.e., transvascular transport models. A continuum approximate poroelasticity model is developed to simulate the interstitial fluid flow in soft subcutaneous tissue. In this section, the governing equations for fluid flow and drug solute transport in the interstitial space and across the vessel network are summarized.

### 2.1. Poroelastic Tissue Model

An approximate poroelasticity model is developed to include the elasticity and compressibility of the skin tissue. This method is both simple and efficient, making it easy to implement. The computational cost is reduced since the constitutive equation to obtain the displacement of a solid skeleton is not solved. At the same time, it can well capture the relaxation of pressure as accurately as the full poroelasticity model [14]. Here, we show the derivation of the approximate poroelasticity model from the Biot poroelasticity theory [32,33,34]. The total stress τ of the poroelastic medium is the sum of the effective stress of the solid skeleton (responsible for the deformation of the solid part of the tissue) and the local fluid pressure as follows:

(1) τ=σ−αpI,

where σ represents the components of effective stress applied to the solid part of the medium, *p* represents the pore pressure applied to the fluid, and α is the Biot’s coefficient. For an incompressible fluid, α=1. Here, I is the identity matrix. Using the force balance, ∇·τ=0, we have:

(2) ∇·œ−α∇p=0.

Based on Biot’s poroelasticity theory [32,34], one form of governing equation for the fluid flow in the poroelastic medium is [14,34,35]:

(3) $$\alpha \frac{\partial \varepsilon_{v}}{\partial t}+\frac{1}{M}\frac{\partial p}{\partial t}=\nabla \cdot \left(\frac{\kappa }{\mu }\nabla p\right),$$

where κ is the permeability of the porous medium μ is the viscosity of the fluid, $\varepsilon_{v}$ is the volumetric strain and *M* is the bulk modulus of the fluid-solid mixture.

For the approximate poroelasticity model, we assume that solid displacement is small, the Darcy velocity of the interstitial fluid is small, and the non-zero component of the solid displacement ı is mainly in the normal direction. Based on our homogeneous and small strain assumption (linear elasticity), we have σ=Eε in one dimension, i.e., Hooke’s law for the elastic response, where *E* is Young’s modulus of the elasticity. For a 3-D simulation, the volumetric dilation is $\varepsilon_{v}=\sum \varepsilon_{i}=\partial {ı}_{i}/\partial x_{i}$ [36], where ı is the displacement of the solid. We have the mean stress $\bar{œ}=\sigma_{ii}/3=E_{v}\varepsilon_{ii}=E_{v}\varepsilon_{v}$, where $E_{v}=E/3\left(1-2\nu \right)$. After applying the stress balance ($E_{v}\varepsilon_{v}=\alpha p$), we obtain the approximation for the time derivative of the volumetric strain $\varepsilon_{v}$ as below:

(4) $$\frac{\partial \varepsilon_{v}}{\partial t}=\frac{\partial \nabla \cdot ı}{\partial t}=\frac{\alpha }{E_{v}}\frac{\partial p}{\partial t}.$$

Finally, the pressure equation for the poroelastic tissue becomes:

(5) $$\left(\frac{\alpha }{E_{v}}+\frac{1}{M}\right)\frac{\partial p}{\partial t}-\nabla \cdot \left(\frac{\kappa }{\mu }\nabla p\right)=0.$$

The mass balance in the interstitial space with solid displacement ı reads as [21],

(6) $$\nabla \cdot \dot{ı}-\nabla \cdot \left(\frac{\kappa }{\mu }\nabla p\right)=0,$$

where $\dot{ı}$ is the time derivative of deformation $\dot{ı}=\partial ı/\partial t$, i.e., the velocity of the solid skeleton $u_{s}$. We define $1/D_{e}=\alpha /E_{v}+1/M$, where $D_{e}$ indicates the general softness of the tissue. After combining Equations (5) and (6), we have the governing equation for the approximate deformation:

(7) $$\nabla \cdot \nabla \zeta -\frac{1}{D_{e}}\nabla p=0.$$

The details of the injection model and the qualification of the proposed continuum poroelasticity model can be found in Appendix A.1 and Appendix A.2, respectively. When utilizing 96-processors parallel computation, it takes approximately one day to complete a simulation using the approximate poroelasticity model, which models the transport and pressure evolution in a poroelastic tissue up to 70 s after the start of the injection. The efficiency of our proposed model is comparable to Darcy’s law model for the porous medium [11]. However, it takes three days to finish the same simulation solving the constitutive equation of poroelasticity [12]. The approximate poroelasticity model improves the computational efficiency by three times compared to the full poroelasticity model.

### 2.2. Transport Equations

A comprehensive model for the extravascular transport of fluid and drug solutes and the transvascular exchange in the tissue is described for both the continuum medium over the length scale of *O* (1 cm) and the discrete vessels over the length of *O* (100 μm) in this Section [11,13].

#### 2.2.1. Fluid Flow in the Interstitial Space

First, Darcy’s law is used to describe the fluid flow in the porous media:

(8) $$u_{D}=-\frac{k}{\mu }\nabla p,$$

where $u_{D}$ is the average velocity of interstitial fluid flow in the porous medium, and $u_{D}=u_{f}-u_{s}$, where $u_{f}$ is the fluid velocity in the pore. On the right-hand side (RHS) of Equation (8), *p* is the interstitial pressure and μ is the dynamic viscosity of the interstitial fluid.

With the flux gain from blood vessels and drainage from lymphatics included, the continuity equation considering the poroelasticity gives [1,37,38,39,40]:

(9) $$\nabla \cdot u_{D}+\nabla \cdot \dot{ı}=q_{r}+J_{v}-J_{l},$$

where $J_{v}$ and $J_{l}$ are the net fluid flux gain from blood vessels and net fluid flux loss to lymphatic vessels per tissue unit volume, respectively, and $q_{r}$ is the source term, representing the injection. For the fluid flow in the proposed approximate poroelastic equation, it satisfies Equations (5) and (6), i.e., $\nabla \cdot \dot{ı}=1/D_{e}\partial p/\partial t$. The fluid conservation equation for a poroelastic medium without any source term could be written as [5,41]. After substituting Equation (8) and the expression for $\nabla \cdot \dot{ı}$ into Equation (9), we have the governing equation for pressure:

(10) $$\frac{1}{D_{e}}\frac{dp}{dt}-\nabla \cdot \left(\frac{k}{\mu }\nabla p\right)=\left(q_{r}+J_{v}-J_{l}\right).$$

The net fluid fluxes through the blood and lymphatic vessels are determined by Starling’s law, which read [37,38,39,40]:

(11) $$\begin{array}{cc} J_{v} & =L_{pv}\frac{S}{V}\left(p_{v}-p_{i}-\sigma \left(\pi_{v}-\pi_{i}\right)\right), \end{array}$$

(12) $$\begin{array}{cc} J_{l} & =L_{pl}\frac{S}{V}\left(p_{i}-p_{l}\right), \end{array}$$

where $L_{pb}$ and $L_{pl}$ are the hydraulic conductivity of blood and lymphatic vessels, respectively. Additionally, S/V denotes the surface area of vessels to the unit volume of tissue. Pressures in blood, lymphatics, and the interstitial space are $p_{v}$, $p_{l}$, and $p_{i}$, respectively. In Equation (11), σ is the osmotic reflection coefficient of blood capillary for the solute, where $\pi_{v}$ and $\pi_{i}$ are the osmotic pressures of the plasma in the blood vessel and the interstitial space, respectively.

#### 2.2.2. Solute Transport in the Interstitial Space

A general solute transport equation at the macroscopic scale is used for the continuum model [11,13,37,38,42]:

(13) $$\frac{\partial \varepsilon C}{\partial t}+\nabla \cdot \left(u_{D}C\right)=\nabla \cdot \left(D\varepsilon \nabla C_{i}\right)+q_{r}+\phi_{b}-\phi_{e}-\phi_{l},$$

where *C* is the dimensionless drug concentration in the interstitium, normalized by the initial concentration $C_{0}$. The effective molecular diffusion coefficient in the interstitial space is denoted as *D*. On the right-hand side, $\phi_{e}$ denotes the elimination of the drug per tissue unit volume due to binding to the ECM and cells, cell uptake, or chemical reaction, $\phi_{b}$ denotes drug perfusion from blood vessels, and $\phi_{l}$ denotes drug drainage to lymphatic vessels. Drug absorption through lymphatic vessels is given by $\phi_{l}=J_{l}C_{i}$ [38,42,43]. In Equation (13), $\phi_{b}$ is set to be zero, considering that large molecules cannot directly transport into blood vessels. The elimination term ($\phi_{e}$) is set to be zero except in the section studying the role of binding and metabolism to focus on the transport and absorption of free drug molecules.

#### 2.2.3. Transvascular Fluid Flow and Solute Transport

The well-known model for solute transport across the membrane, the Kedem–Katchalsky (K-K) formulation, is used for solute transport across the blood vessels. The fluid and solute fluxes are expressed as:

(14) $$\begin{array}{cc} J_{B}{|}_{\partial \Omega_{B}} & =L_{pb}\varepsilon \left(p_{b}-p_{i}-\sigma_{b}\left(\pi_{b}-\pi_{i}\right)\right), \end{array}$$

(15) $$\begin{array}{cc} j_{bs}{|}_{\partial \Omega_{B}} & =J_{B}{|}_{\partial \Omega_{B}}\left(1-\sigma_{b}\right)\left(C+C_{b}\right)/2+P_{b}^{\alpha }\Delta C, \end{array}$$

where $J_{B}$ denotes the fluid flux and $j_{bs}$ denotes the solute flux per unit area across the blood capillary wall consisting of the convective and diffusive transport of solute across the vessel wall (i.e., membrane). The convective transport of solute is determined by the convective fluid flow $J_{B}{|}_{\partial \Omega_{B}}$, while the diffusive transport of solute is determined by the solute permeability $P_{b}^{\alpha }$. The average concentration across the membrane is calculated by $(C+C_{b})/2$, where $C_{b}$ is the concentration of drug solution inside the blood vessels and *C* is the concentration in the interstitial space. Here, all concentrations are normalized by the initial concentration of the injected drug solution $C_{0}$. The diffusive transport is determined by the difference between the concentration in the interstitial space and the blood vessels ΔC, which is calculated by $C-C_{b}$. The reflection coefficient σ and the solute permeability $P_{b}$ are used to take into account the permeability of the blood vessel membrane for various solutes. Considering that large molecules (such as monoclonal antibodies with molecular weight over 150 kPa) cannot transport through the blood vessels, we have σ=1 and $P_{b}=0$ to represent a semipermeable membrane [13].

**Figure 1:**
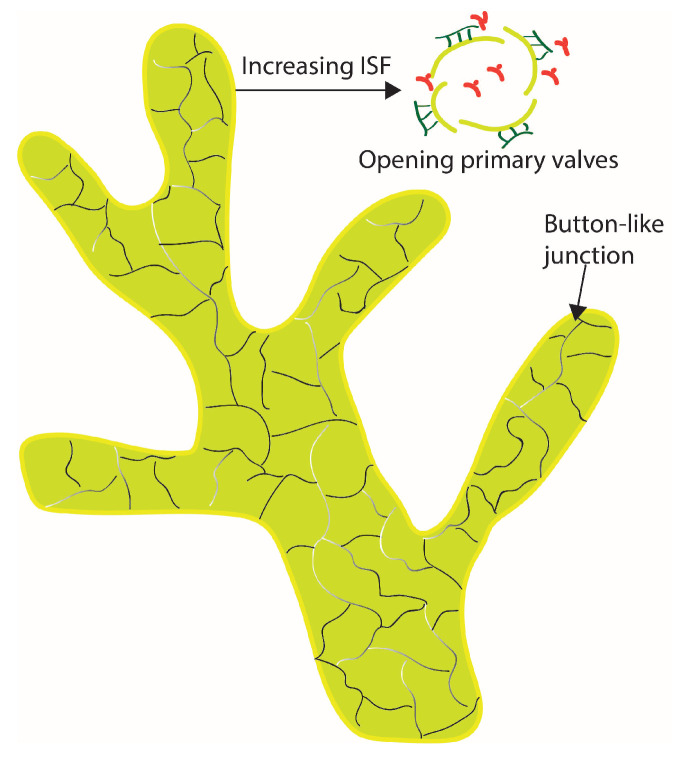
Illustration of a small branch of initial lymphatics.

The lymphatic vessel consists of one single layer of endothelial cells lacking the basement membrane and thus, an improved Kedem–Katchalsky (iK-K) model is developed to describe the transport of macromolecules across the lymphatic vessel wall. The fluid flux $J_{L}$ and solute flux $j_{ls}$ across the lymphatic vessel wall are:

(16) $$\begin{array}{cc} J_{L}{|}_{\partial \Omega_{L}} & =\delta_{valve}L_{pl}\varepsilon \left(p_{i}-p_{l}\right), \end{array}$$

(17) $$\begin{array}{cc} j_{ls}{|}_{\partial \Omega_{L}} & =J_{L}{|}_{\partial \Omega_{L}}\left(1-\sigma_{l}\right)\left(C_{l}+C\right)/2+P_{valve}\left(C-C_{l}\right). \end{array}$$

where $\delta_{valve}$ is used to represent the primary valve structure of the initial lymphatics, where slits are opened when the interstitial pressure (ISF) $p_{i}$ is larger than the pressure inside the lymphatics $p_{l}$. The primary valves openings allow for the transport of large molecules into initial lymphatics, as shown in Figure 1. Solute permeability through the valve $P_{valve}$ is related to the partition coefficient of the solute Φ, which is a function of the Stokes radius of the solute, primary valve opening area fraction $\delta_{f}$, and diffusion across the opening slit $D_{valve}$.

(18) $$\begin{array}{cc} P_{valve} & =\delta_{f}D_{valve}\Phi \left(r_{s}\right)/d_{l}, \end{array}$$

(19) $$\begin{array}{cc} \Phi (r_{s}) & =\delta_{valve}^{\prime }\left(1-\frac{r_{s}}{w/2}\right). \end{array}$$

where $\delta_{f}$ is the primary valve opening area fraction, which is the ratio of the area of the valve opening slit and the area of the lymphatic vessel $A_{valve}/A_{vessel}$ per unit length, where $A_{valve}/A_{vessel}=f\times L_{l}\times w$. Here, *w* is the opening width, $L_{l}$ is the length of the slit per unit area of the lymphatic vessel wall, and *f* is the fraction of slit length open to valve width *w*. $\Phi (r_{s})$ is the solute partition coefficient, which is a function of the solute Stokes radius, where $\delta^{\prime }$ is used to ensure the partition coefficient is positive. $\delta^{\prime }=0$ when half the width of the opening valve is smaller than the Stokes radius of the solute ($w/2<R_{s}$) and $\delta^{\prime }=1$ when $w/2>R_{s}$. The thickness of the lymphatic membrane is denoted as $d_{l}$. We use an exponential function to represent the variation of opening width *w* with the pressure difference between the interstitial pressure and the lymphatics $\Delta p=p_{i}-p_{l}$ [26,44].

(20) $$w=w_{0}+w_{1}\frac{1}{1+e^{5-100\Delta p}}.$$

where the pressure difference Δp is in unit kPa. The opening width of the primary valves on the lymphatic vessels *w* is 10∼40 nm at the basal pressure and the width of the opening slit can reach as high as 64nm at high interstitial pressure due to the injection according to the simulations in [44]. The baseline values for the parameters in the improved Kedem–Katchalsky model are shown in Table 1 [26,27,44,45].

**Figure 2:**
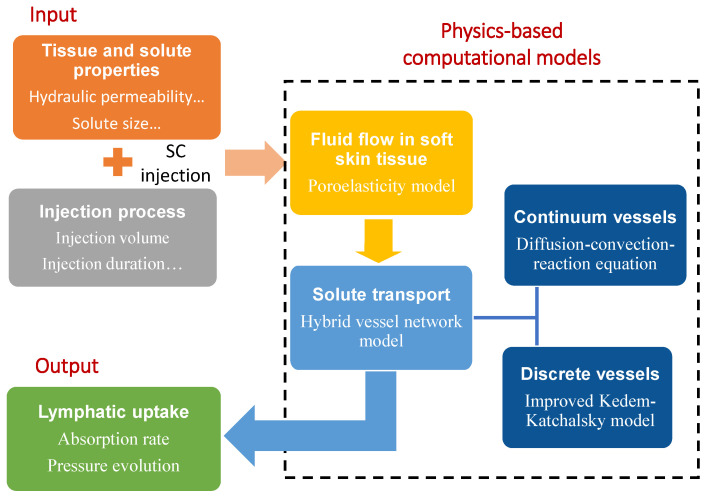
Conceptual overview of the physics-based computational models for lymphatic uptake in soft skin tissue through subcutaneous injection.

The tissue and solute properties (such as hydraulic permeability and solute size) and injection process parameters (such as injection duration and volume) are inputs needed for the computational model, as shown in Figure 2. The fluid flow in soft porous tissue is modeled using the proposed poroelasticity model. The solute transport is modeled by the hybrid vessel network model. In the hybrid vessel network model, the diffusion–convection–reaction equation is used to simulate solute transport through the continuum vessels. On the other hand, the improved Kedem–Katchalsky model is developed to simulate the solute transport across the lymphatic vessel membrane for the discrete vessels. With these physics–based computational models as shown in Figure 2, we can predict the absorption rate and evolution of the interstitial fluid pressure [48].

### 2.3. Computational Setup

The computational domain for the continuum model without explicit vessel network is 80×80×50mm, while the computational domain in the case with explicit vessel network is 50×50×50mm, including epidermis, dermis, subcutaneous tissue, and muscle layers [49]. The 80×80×50mm domain is set to be a symmetric one to allow for a large injection volume (2 mL), where only a quarter of the whole injection site is simulated, with a symmetric boundary condition on the symmetry plane. The injection point $P_{0}$ (center of the injection bolus) is located 4mm below the top surface along the symmetry axis in the single-layered domain, while the injection depth is 5mm in the multi-layered domain. Unless otherwise specified, the injection volume is 1 mL for all simulations in this paper. The baseline values for all physical and physiological parameters used in the computational model are listed in Table 2, which are of the same order of magnitude as the previous studies [11,13,38,42,50,51,52], and the units are all SI base units.

The mesh is refined near the injection point and the smallest mesh size used is h=0.1R, where *R* is the characteristic length, equal to the radius of the injection bolus, and time step Δt=0.001s. Based on the mesh convergence study, we choose the second good mesh (256×256×256) and after the local refinement near the explicit vessel walls, the total number of cells is around 31 million, through which we can accurately and efficiently resolve the solute transport across the lymphatic vessels.

The governing equations and computational models are implemented and solved based on the open-source Computational Fluid Dynamics platform, OpenFOAM. The boundary conditions and numerical schemes for the numerical simulation can be found in Appendix A. The key inputs for the model are the tissue and solute transport properties as well as injection process parameters: hydraulic permeability of the porous skin tissue *k*, elasticity modulus of the soft tissue E, the diffusivity of the drug solute in the ECM *D* and across the vessel membrane $D_{valve}$, vascular permeability of blood capillaries $L_{pb}$, vascular permeability of lymphatic capillaries $L_{pl}$, the Stokes radius of the drug solute $r_{s}$, implicit vessel network density S/V, injection volume, injection duration, and injection depth. The values of these parameters are listed in Table 1 and Table 2.

## 3. Pressure Build-Up and Relaxation

Interstitial fluid pressure (IFP) plays a crucial role in regulating fluid flow and mass transport in the SC tissue. The pressure build-up process in the soft subcutaneous tissue is important to understand the mechanism of drug transport and absorption. The fluid flow and solute transport across the lymphatic vessels and blood vessels are governed by the pressure in the interstitial tissue and vessels, as shown in Equations (11) and (12). Here, the deformable subcutaneous tissue is expressed as a poroelastic medium using the approximate poroelasticity model. In this section, we investigate the effect of various elasticity and permeability, variable permeability, and the heterogeneity of elasticity and permeability in the multi-layered tissue on the pressure build-up during and after the injection process. Absorption and hybrid vessels are not included in these cases.

### 3.1. Elasticity and Permeability

First, we study the effect of elasticity on the development of pressure in subcutaneous tissue. The young’s modulus of skin is affected by many factors, such as age, gender, hydration, and skin thickness [53]. The young’s modulus or elastic modulus *E* varies a lot for different layers of the tissue, as shown in Table 3. The young’s modulus *E* of subcutaneous tissue, which is also known as the hypodermis, is around 2 kPa according to [54]. Here, we study how the elastic modulus *E* affects the pressure build-up in soft and deformable tissue compared to rigid porous tissue modeled by Darcy’s law over a wide range of *E*, assuming the isotropic elasticity in the subcutaneous tissue.

**Figure 3:**
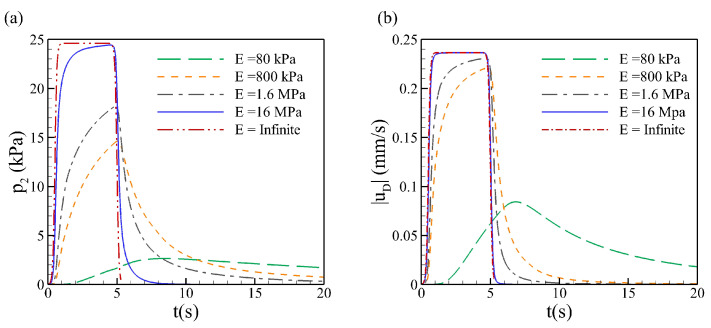
Time evolution of (**a**) interstitial fluid pressure $p_{2}$, (**b**) magnitude of Darcy velocity $|u_{D}|$, at the point $P_{2}$, 10mm below the injection point, for various elastic modules *E*. For a rigid porous medium modeled by Darcy’s law E→∞.

From Figure 3, we can see that with smaller elasticity *E*, the relaxation time is larger, which is proportional to 1/E, and it takes a longer time for the pressure to reach zero and has a lower peak. For a rigid porous medium (E→∞), which is modeled by Darcy’s law, the pressure drops immediately at the end of the injection process t=5s. The same sudden decrease happens for the velocity. As the material gets softer with smaller elasticity, the material absorbs the kinematic energy leading to lower pressure and velocity and elongated relaxation time. The interstitial fluid pressure relaxes to a small value, as both solid and fluid phases are incompressible, and the dilatation only happens during the injection [21].

**Figure 4:**
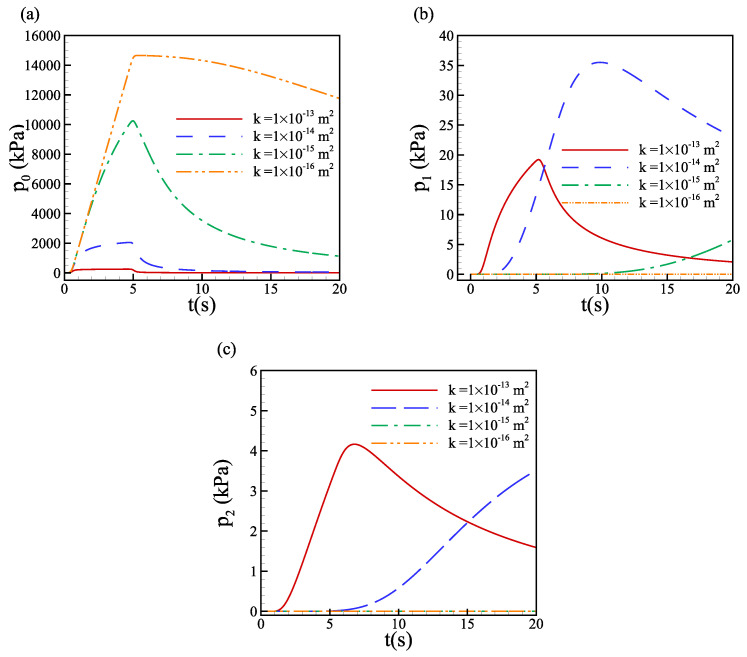
Time evolution of interstitial fluid pressure at (**a**) the injection point $P_{0}$, (**b**) point 2 mm below the injection point $P_{1}$, (**c**) point 10 mm below the injection point $P_{2}$, along the symmetric axis of the domain (center line), for various hydraulic permeability *k*. The elastic modulus E=80 kPa.

For tissues from different parts of the human body and different animals, the hydraulic conductivity *K* and the hydraulic permeability *k* (K=k/μ) vary over a wide range [11,55,57,58,59]. The magnitude of permeability *k* varies over a wide range from $10^{-16}\;m^{2}$ to $10^{-13}\;m^{2}$ in the literature. Pressure evolution at three points, i.e., $P_{0}$, $P_{1}$ and $P_{2}$ along the symmetric axis of the domain, denoted as $p_{0}$, $p_{1}$ and $p_{2}$, respectively, are compared for different permeability values in the poroelastic tissue, as shown in Figure 4. With smaller permeability, the pressure peak at point $P_{0}$ increases dramatically from about 200 kPa ($k=1\times 10^{-13}\;m^{2}$) to 2000 kPa ($k=1\times 10^{-14}\;m^{2}$), and for $k=1\times 10^{-15}\;m^{2}$ and $k=1\times 10^{-16}\;m^{2}$, the peak pressures are 10 Mpa and 14.5 Mpa, respectively, as shown in Figure 4a. Although the magnitude of pressure for different permeability significantly changes, the pressure at the injection point reaches the maximum value almost at the same time, i.e., at the end of the injection. With the decrease of permeability *k* and increase of the magnitude of pressure, the relaxation time increases dramatically at fixed elasticity E=80 kPa. For $k=1\times 10^{-13}\;m^{2}$, the pressure $p_{0}$ relaxes to zero quickly after the injection, while $p_{0}$ for the tissue with $k=1\times 10^{-16}\;m^{2}$ is still large at the end of observation time t=20s. For the other two points, the pressure profile is different for different permeability values. As shown in Figure 4b, the pressure increases at a lower rate $P_{1}$ for $k=1\times 10^{-14}\;m^{2}$, and much lower for $k=1\times 10^{-15}\;m^{2}$ compared to $k=1\times 10^{-13}\;m^{2}$. At point $P_{2}$, the pressure for $k=1\times 10^{-15}\;m^{2}$ remains almost zero within 20s, as shown in figure Figure 4c. This is because the fluid can not spread as much farther from the injection point as the permeability decreases to a very small value. Due to the accumulation of fluid, the interstitial pressure $P_{0}$ is larger at the injection point for low permeability values.

Hydraulic permeability plays a key role in the magnitude of the pressure. Specifically, the tissue deformation is related to the hydraulic permeability of the porous medium as well as the viscosity of the fluid. When the permeability is rather small, the magnitude of pressure is very large, and a large elastic modulus of the same magnitude as the pressure can significantly affect the pressure evolution. For instance, for $k=1\times 10^{-16}\;m^{2}$, the peak pressure is about the order of *O* (1MPa) when the elastic modulus *E* reaches *O* (1MPa). The evolution of pressure shows a prominent difference compared to rigid porous media with E→∞, and the relaxation time increases dramatically with decreasing elasticity. When the hydraulic permeability is larger, the pressure magnitude is smaller and it is more sensitive to small elasticity values of the tissue. For instance, for the case with $k=1\times 10^{-13}\;m^{2}$, the pressure profile changes dramatically when the elastic modulus is as small as *O* (1kPa) while it shows little difference compared to rigid porous media when the elastic modulus is of order *O* (1MPa).

### 3.2. Variable Porosity and Permeability

The deformation of soft tissue leads to a variable fluid volume fraction or porosity in the tissue, which can be described by Equation (21). Porosity ε, here, is not uniform and constant in this case, and it varies depending on the tissue deformation. The evolution of porosity ε could be described by Equation (21), as shown in the following equation:

(21) $$\varepsilon =1-\frac{1-\varepsilon_{0}}{det(I+\nabla D)},$$

where we have the gradient of deformation $\nabla D=(1/M+\alpha /E_{v})p$ for the proposed poroelastic tissue model. The hydraulic permeability of the tissue depends on the properties of the tissue matrix, including the porosity and fiber spacing [57]. Various forms of permeability *k* dependence on the strain have been proposed in the literature [60]. Here, we adopt the Kozeny–Carman (equation Equation (22)) to account for the spatially variable permeability as a function of porosity, which intrinsically depends on pressure. Variable porosity and permeability have been assumed to be a function of pressure, which should be experimentally determined [61]. The domain size used here is the same as the validation section, which is 80×80×100mm, and we focus on the effect of porosity-dependent permeability.

(22) $$\kappa \left(\varepsilon \right)=\kappa_{0}\frac{{\left(1-\varepsilon_{0}\right)}^{2}}{\varepsilon_{0}^{3}}\frac{\varepsilon^{3}}{{\left(1-\varepsilon \right)}^{2}}$$

**Figure 5:**
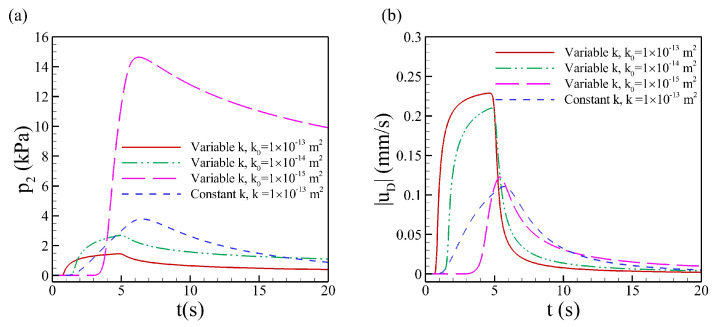
Time evolution of (**a**) interstitial fluid pressure $p_{2}$ and (**b**) magnitude of Darcy velocity $|u_{D}|$ for variable permeability compared to constant permeability. The pressure and velocity are results at the point $P_{2}$, 10mm below the injection point. The initial porosity ε=0.01 and elasticity $E_{v}=80$ kPa.

**Figure 6:**
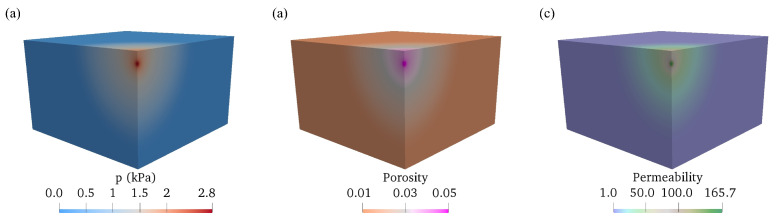
Distribution of (**a**) interstitial fluid pressure p with values in unit of kPa, (**b**) porosity, (**c**) hydraulic permeability with values in unit of $10^{-13}\;m^{2}$ at the end of injection t=5.0s in the single-layered domain with variable porosity and permeability. The initial porosity, $\varepsilon_{0}=0.01$, and permeability, $k_{0}=1\times 10^{-13}\;m^{2}$.

The time evolution of the pressure *p* and Darcy velocity $u_{D}$ are compared for porosity-dependent permeability and constant permeability values, as shown in Figure 5. The initial value of porosity $\varepsilon_{0}$ is set to be 0.01, and permeability $k_{0}$ varies in a range from $1\times 10^{-15}\;m^{2}$ to $1\times 10^{-15}\;m^{2}$, respectively. For a constant permeability case, $k=1\times 10^{-13}\;m^{2}$. During the injection process, permeability changes with pressure, following Equation (21), and the hydraulic permeability *k* of the porous medium varies following Equation (22). As shown in Figure 6a,b, the magnitude of the maximum pressure for variable permeability with $k_{0}=1\times 10^{-13}\;m^{2}$ is less than half that of constant permeability. The velocity behaves the opposite; the maximum velocity of variable permeability is twice that of constant porosity and permeability. With smaller initial permeability, the pressure peak increases, and velocity decreases. Especially for $k=1\times 10^{-15}\;m^{2}$, the pressure peak rapidly reaches a much higher value.

The distribution of permeability and porosity in a single-layered domain is displayed to illustrate the effect of the injection. As shown in Figure 6, with increasing pressure due to the injection of fluid, the porosity increase due to the deformation of the tissue. The increasing porosity leads to a higher permeability. Porosity ε and permeability *k* are much larger near the injection point, as shown in Figure 6b,c. The increasing permeability helps mitigate the large fluid pressure at the injection point, where fluids could permeate through the tissue and spread out more easily.

The interstitial fluid pressure inside the tissues can be easily affected by the measurement and experimental equipment [17,62,63]. There are very limited quantitative measurements or biological data on the dynamic variation of pressure [17]. The evolution of pressure obtained from the in vivo porcine tissue injection in [17] shows similar profiles as in our simulations.

## 4. Transport and Lymphatic Uptake of Drug

In this section, the effect of the heterogeneity of tissue properties in different layers and the heterogeneous vessel network with different structures on the transport and absorption of drug molecules are investigated in poroelastic tissue with variable porosity and permeability. Starting from this section, the drug absorption and the hybrid vessel networks are included.

### 4.1. Multi-Layered Tissue with an Implicit Vessel Network

A multi-layered computational domain is used in this section to make the simulation closer to the real tissue. As shown in Table 3, the elasticity for different skin layers varies in a wide range. In our model, the elasticity for the epidermis, dermis, subcutaneous tissue, and muscle layers is set to be 35 kPa, 10 kPa, and 80 kPa, respectively. Based on a previous study on the thickness of skin layers [49], the thickness of different skin layers varies by many factors, such as the location in the body, gender, and hydration. Here, we use the mean values of the abdomen and thigh for each skin layer. The thickness of the dermis and subcutaneous layers is 2.2 mm and 13.9 mm, respectively, and the muscle layer thickness is set to be 19.9 mm. The hydraulic permeability for each layer varies from $\kappa_{0}=1\times 10^{-16}\;m^{2}$ for the dermis layer to $\kappa_{0}=1.0\times 10^{-13}\;m^{2}$ for the subcutaneous tissue layer [57], as listed in Table 4. We first investigate the drug transport and the lymphatic uptake through the implicit vessel network in a multi-layered poroelastic domain, where surface area per unit volume S/V (implicit vessel network) varies in different layers.

**Figure 7:**
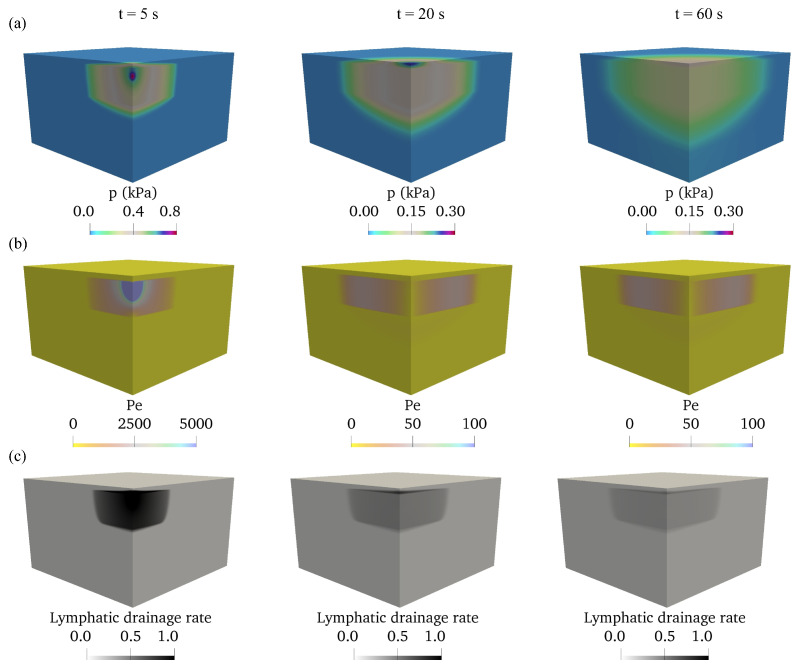
Distribution of (**a**) interstitial fluid pressure *p* with values in unit of kPa, (**b**) Péclet number Pe and (**c**) lymphatic drainage $\phi_{l}$ with values in unit of $10^{-6}\;s^{-1}$, in a multi-layered domain at t=5s, 20s, 60s.

The layer-specific mechanical properties affect the pressure build-up and relaxation in soft tissues [12,14,20]. To understand the convective transport driven by the pressure in the interstitial space for the continuum model, the spatial distribution of interstitial fluid pressure, Reynolds number, and the absorption due to lymphatic drainage are displayed at different moments in Figure 7. The interstitial pressure peaks at the injection point and the magnitude of interstitial pressure decreases gradually with time, as shown in Figure 7a. The Péclet number (Pe) represents the ratio of convective and diffusive transport. The distribution of the Péclet number in the domain is used to indicate the convective transport driven by the interstitial pressure (ISF). The convective transport from the interstitial space into the lymphatic vessels is stronger due to the large interstitial pressure during the injection. At the end of the injection t=5s, Pe reaches *O* $\left(10^{4}\right)$, where the convection is much stronger than the diffusion in the continuum domain. After the injection, the convection gradually decreases as the interstitial pressure decreases to *O* (100Pa) at t = 20 s and t = 60 s. The convective transport of fluid under pressure difference leads to lymphatic uptake. The lymphatic drainage is strong under high interstitial pressure; however, the injection duration is short. As shown in Figure 7c, the magnitude of lymphatic drainage decreases from $1\times 10^{-5}\;s^{-1}$ at t = 5s to $1\times 10^{-7}\;s^{-1}$ at t=60s.

### 4.2. Multi-Layered Tissue with a Hybrid Vessel Network

The injection leads to increased interstitial pressure which gradually relaxes after the injection, as shown in Figure 6 and Figure 7 in previous sections. The pressure difference between the interstitial space and the lymphatic vessel leads to a convective fluid flow into the lymphatic vessels. The evolution of interstitial fluid pressure is affected by mechanical properties, such as elasticity and permeability, as shown in previous sections. In this section, we study the convective transport and absorption of drug molecules through a 3D hybrid discrete-continuum vessel network in multi-layered soft skin tissue. The details about the generation of the explicit vessel network and the corresponding computational setup can be found in our previous papers [11,13].

#### 4.2.1. Improved Kedem–Katchalsky Model

**Figure 8:**
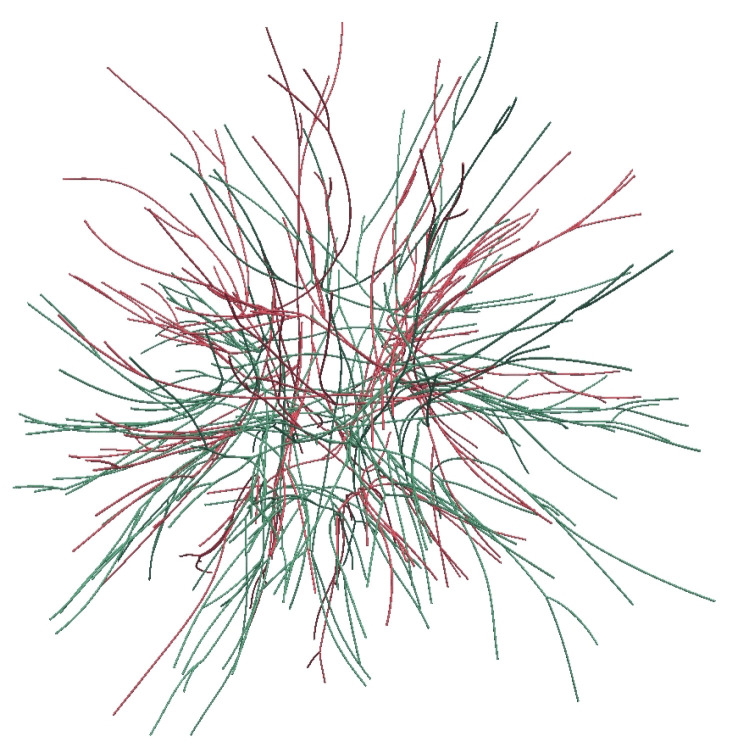
Vessel network of blood (in red) and lymphatic capillaries (in green).

To illustrate the advantage of the improved Kedem–Katchalsky model in addressing the microscopic properties of the lymphatic membrane, we investigate the convection and diffusion of various molecules with different molecular weights and diffusivity. The hydrodynamic radius of a monomeric immunoglobulin with a molecular weight of 15 kDa is normally around 5 to 6 nm [64]. For albumin with a molecular weight of 66 kDa, the Stokes radius is 3.3 nm [47], and the solute diffusivity is around $5\times 10^{-11}\;m^{2}/s$ [65]. For proteins with molecular weight around 1000 kDa, the Stokes radius is around 9 nm. In this section, 0.2mL drug solution is injected into the cubic domain (50×50×50mm). A discrete vessel network for blood vessels and lymphatic vessels with uniform radius $r_{v}=80\;\mathrm{nm}$, as shown in Figure 8, is embedded into the multi-layered domain. We investigate the transport of various drug solutes by varying Stokes radius $r_{s}$ from 3 nm to 9 nm and the solute diffusivity across the lymphatic valve $D_{valve}$ from $5\times 10^{-11}$ to $1\times 10^{-12}\;m^{2}/s$.

The solute diffusion across the lymphatic endothelial cells is significantly affected by the convective fluid flow into the lymphatics because the opening of the primary valves on the lymphatic vessel wall is determined by the pressure difference, i.e., the convective flow. The diffusion through the opening slits on the lymphatic vessels is also affected the convective lymphatic flow inside the lumen (rapid lymphatic drainage), and thus, it is called convective diffusion here.

The convective and diffusive transport across the lymphatic vessel is analyzed here through the local Péclet number, $Pe_{l}$. Here, $Pe_{l}$ is defined as $U_{l}L/D_{valve}=U_{l}/P_{valve}$, where $U_{l}$ is the velocity across the lymphatic vessel wall. The magnitude of $U_{l}$ can be evaluated as $U_{l}∼\varepsilon L_{pl}\Delta p$, which is *O* $\left(10^{-10}\;m/s\right)$ at the end of the injection. The solute diffusivity across the lymphatic vessel $D_{valve}$ divided by characteristic length scale *L* can be evaluated by the solute permeability $P_{valve}$, the magnitude of which is *O* $\left(10^{-9}\;m/s\right)$ for the baseline case with $D_{valve}=10^{-11}\;m^{2}/s$. Thus, the local Péclet number is 1 for the baseline case representing the diffusive and convective transport of solute are of the same magnitude. For the solute diffusivity $D_{valve}=10^{-12}\;m^{2}/s$, the ratio of convective and diffusive transport is 1.

**Figure 9:**
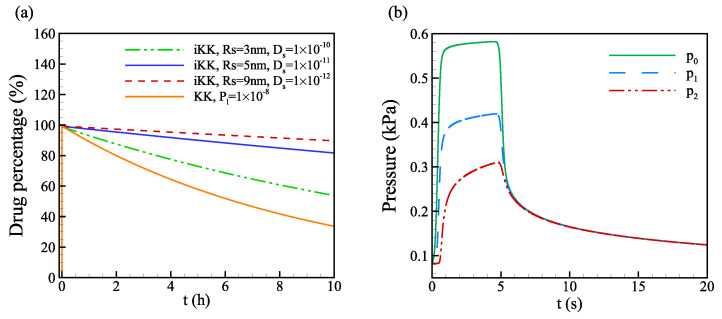
(**a**) Time evolution of drug percentage in the tissue (solute volume normalized with the initial injection volume) for various Stokes radius $r_{s}$ and solute diffusivity across the discrete lymphatic vessel wall. (**b**) Time evolution of interstitial fluid pressure $p_{0}$ (the injection point), $p_{1}$ (2 mm above the injection point), and $p_{2}$ (5 mm below the injection point) at three different points along the symmetric axis of the domain for the baseline case (Rs=5nm).

The evolution of normalized drug volume, i.e., drug percentage, in the tissue for various molecules with different Stokes radii $r_{s}$ and solute diffusivity $D_{s}$ is compared in Figure 9a. The absorption rate is larger for drugs with smaller Stokes radii and larger diffusivity. The characteristic exponential decay time $\tau_{d}$ (i.e., time reaches characteristic time for exponential decay, drug volume reaches 36.8% of the initial volume $V=e^{-1}V_{0}=36.8\%\;V_{0}$) within 300 s is used to indicate the drug absorption rates [13], which are 16 h, 51 h, and 98 h for Rs=3nm with $D_{s}=1\;\times 10^{-10}\;m^{2}/s$, Rs=5nm with $D_{s}=1\times 10^{-11}\;m^{2}/s$ and Rs=9nm with $D_{s}=1\times 10^{-12}\;m^{2}/s$, respectively. At the same convective flow, the pressure difference is the same between the interstitial space and the lymphatics. However, the solute permeability for smaller solutes is higher as the larger partition coefficient due to the opening of the primary valves and the solute diffusivity is larger, leading to a higher absorption rate. To illustrate the effects of the larger partition coefficient and diffusivity due to decreasing Stokes radii separately, the drug characteristic exponential decay time within 10 s for various solute sizes with fixed diffusivity and different diffusivity are compared in Table 5. In the short term, the effect of solute diffusivity coming from the change of the Stokes Radius is more prominent than the pure change of the Stokes Radius (i.e., the change of partition coefficient). For Rs=9nm, $\tau_{d}$ increases to 10.3 h compared to Rs=5nm with the same diffusivity, causing 18% difference in absorption rate. With decreasing diffusivity $D_{s}=1\times 10^{-12}\;m^{2}/s$ for Rs=9nm, $\tau_{d}$ increases to 48.8 h, causing 81% difference in absorption rate. In the longer term, the effect of solute diffusivity is smaller as the diffusion slows down due to decreasing concentration gradient and interstitial fluid pressure.

Compared to the Kedem–Katchalsky model with only solute permeability $P_{l}^{\alpha }$ determining the diffusion across the lymphatic vessel wall, the improved Kedem–Katchalsky model can take into account the effects from properties of the vessel membrane and solute molecules on the convective diffusion across the opening of the primary valves, such as the solute size and solute diffusivity across the lymphatic opening slits. From Figure 9a, the absorption rate is lower for solute flux governed by the improved Kedem-Katchalsky model than by the Kedem–Katchalsky model with $p_{l}=1\times 10^{-8}\;m/s$.

In the soft interstitial tissue, the pressure gradually decreases after the end of injection (t = 5 s), as shown in Figure 9b. The time evolution of the interstitial pressure at different points in the domain decreases to around 0.12 kPa at t = 20 s. The interstitial pressure relaxes to the resting pressure $p_{\infty }\;\left(82.4\;\mathrm{Pa}\right)$ at around 200 s. Thus, the convective transport and the effect of large interstitial pressure due to the injection in soft skin tissue mainly exist during this period.

#### 4.2.2. Structure of the Lymphatic Vessel Network

The morphology of the lymphatics varies from fractal trees to interconnected irregular shapes depending on the location of biological tissues and species, as reported in [2,3,28,30,66]. In this section, a discrete vessel network for the lymphatic vessels with uniform radius $r_{v}=200\;\mathrm{nm}$ is embedded into a domain of size 50×50×36mm. Blood vessels are implicitly included in the model.

**Figure 10:**
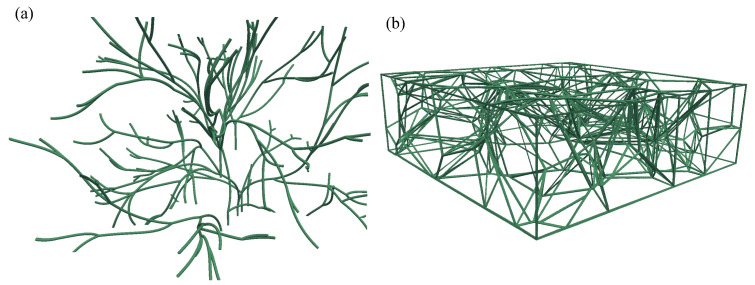
Lymphatic vessel networks with different structures: (**a**) fractal trees, (**b**) Voronoi structure.

First, the fractal tree structure is generated using the space-fill fractal algorithm [67] to represent the branches of the lymphatic vessels, as shown in Figure 10a. In addition to fractal trees, we use the Voronoi structure to mimic the interconnected initial lymphatic vessel network structure, as shown in Figure 10b. The fractal tree structure and Voronoi structure of lymphatics vessels are embedded into the 5×5×3cm domain. Specially, the height of the Voronoi structure is 1.6 cm assuming such a lymphatic structure exists in the dermis and subcutaneous layer. The equivalent surface area of lymphatic vessels per unit volume of the tissue for these two structures is $188.9\;m^{-1}$ and $114.8\;m^{-1}$, respectively.

**Figure 11:**
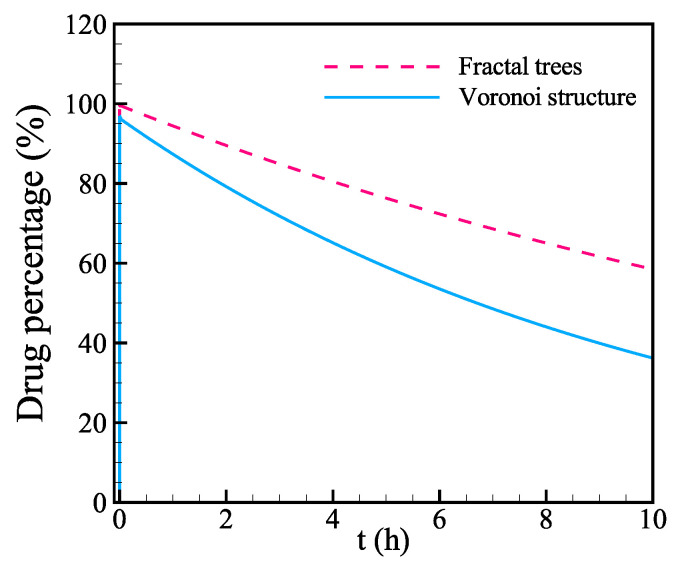
Comparison of drug evolution in the tissue with two different lymphatic vessel network structures.

First, we quantitatively compare the evolution of drug volume in tissue for these two structures. As shown in Figure 11, the absorption is much faster through the Voronoi structure than the fractal trees. At the end of the injection, there is 95.8% of the total drug left in the tissue with Voronoi-structured lymphatic vessel network, while that value is 98.6% for fractal trees. During the 5 s injection, the difference in absorption percentage is around 2.8% for these two structures. The characteristic time within 300 s is 18 h and 10 h, respectively, for these two structures. The absorption through the Voronoi- structured vessel network is significantly faster than the fractal tree structure.

**Figure 12:**
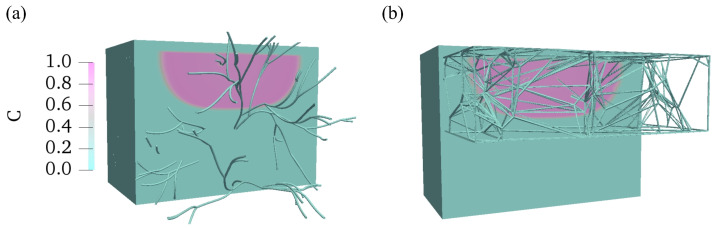
Comparison of drug distribution in multi-layered skin tissue with two different structures, (**a**) fractal trees, (**b**) Voronoi structure, at t = 300 s.

To further illustrate the high absorption efficiency of the Voronoi structure of the lymphatic network, we investigate the drug transport across the explicit vessel wall. The drug distribution is compared in multi-layered tissue with fractal tree-structured and Voronoi-structured lymphatic vessel networks at t = 100 s. As shown in Figure 12, there are more vessels in contact with the drug plume on the central plane of the domain for the Voronoi-structured lymphatic vessel network. The topology of the Voronoi structure leads to accumulated and concentrated vessels in the dermis and subcutaneous layers.

## 5. Conclusions

Interstitial fluid pressure plays a crucial role in governing the convective transport and absorption of drug molecules in the tissue. An approximate continuum poroelasticity model is developed to mimic the pressure build-up and relaxation by introducing the time variation of pressure. The effects of elasticity, porosity, and permeability on pressure evolution are investigated after validating the proposed poroelastic model with the analytical solution and previous computational model. The advantage of this model lies in the simplicity of implementation while maintaining good accuracy in capturing the evolution of the interstitial pressure. The small elasticity of the tissue slows down the relaxation of pressure and lowers the magnitude of pressure, while the magnitude of pressure increases dramatically with decreasing hydraulic permeability. Pressure evolution for low hydraulic permeability tends to be more sensitive to small elasticity, i.e., the relaxation time is longer for low hydraulic permeability at the same elasticity. The effect of variable porosity-dependent permeability is found to alleviate the high pressure due to increased porosity and permeability near the injection site.

To better address the microscopic structure of the lymphatic vessel membrane, an improved Kedem–Katchalsky model is developed for solute transport across the explicit vessel wall. The transport and lymphatic uptake of drug solutes in a multi-layered medium are investigated to elucidate the effect of heterogeneity of multi-layered structure and the heterogeneous vessel network. We investigate the convective diffusion of various macromolecules through the hybrid lymphatic vessel network by varying the Stokes radius and solute diffusivity across the opening lymphatic valves. With a smaller Stokes radius and larger solute diffusivity, the lymphatic uptake is significantly fast. Finally, the effect of the lymphatic vessel network structure is investigated through the fractal trees and Voronoi structures. We find that the Voronoi structure in the subcutaneous layer is more efficient in drug absorption because there are more interactions between the drug molecules and the vessel surfaces in the dermis and hypodermis layers.

For future work, the computational model can be further coupled with the mathematical model for the lymph flow inside the lymphatic vessels. In this way, the effect of the lymph flow rate can be incorporated into the model. The computational models developed here can also be coupled with the pharmacokinetic (PK) models to better predict drug bioavailability.

## Figures and Tables

**Table 1:** Baseline values for parameters used in the improved Kedem-Katchalsky model.

Parameter	Value and Range	Unit	References
Slit length per unit area of vessel wall $L_{l}$	$1\times 10^{5}$	$m/m^{2}$	[27,45]
Valve width $w_{0}$	10	nm	[44]
Valve width $w_{1}$	50	nm	[44]
Fraction of length of slit opening to valve width f	0.5	1	[46]
Diffusion coefficient within a valve $D_{valve}$	$10^{-11}$	$m^{2}/s$	[47]
Solute permeability within a valve $P_{valve}$	$10^{-9}$	m/s	calculated
Lymphatic capillary radius $r_{l}$	200	μm	[2]
Lymphatic capillary membrane thickness $d_{l}$	$5\times 10^{-7}$	m	[26,27]
Stokes radius of solute $r_{s}$	$5\times 10^{-9}$	m	[47]

**Table 2:** Baseline values for various parameters in the computational model.

Parameter	Value and Range	Unit
Hydraulic permeability $k_{0}$	$1\times 10^{-13}$	$m^{2}$
Elastic modulus *E*	80	kPa
Bulk modules *M*	267	kPa
Initial porosity $\phi_{0}$	0.01	1
Retardation factor $R_{f}$	1	1
Diffusion coefficient *D*	1 $\times 10^{-11}$	$m^{2}/s$
Vascular hydraulic conductivity $L_{pl}$	$3.6\times 10^{-11}$	m/(Pa.s)
Vascular hydraulic conductivity $L_{pb}$	$1\times 10^{-12}$	m/(Pa.s)
Reflection coefficient σ	0.3	1
Osmotic pressure $\pi_{v}$	3000	Pa
Osmotic pressure $\pi_{i}$	1500	Pa
Blood vessel pressure $P_{v}$	3500	Pa
Lymphatic pressure $p_{l}$	0	Pa
Surface area per volume S/V	7000	$m^{-1}$

**Table 3:** Elastic modulus *E* of different skin layers and tissues.

Layer	E (Mpa)	Reference
Stratum Corneum	500	[54]
Epidermis	6	[53]
Dermis	8∼35 $\times 10^{-3}$	[55]
Hypodermis	$2\times 10^{-3}$	[54]
Muscle	$80\times 10^{-3}$	[56]

**Table 4:** Mechanical and physiological properties, including elasticity *E*, hydraulic permeability *k*, surface area per unit volume S/V, and thickness of different skin tissue layers.

Layers	*E* (kPa)	*k* (m/s)	S/V ($m^{-1}$)	Thickness (mm)
dermis	35	$1\times 10^{-15}$	5000	2.2
subcutaneous tissue	10	$1\times 10^{-13}$	3000	13.9
muscle	80	$1\times 10^{-14}$	1000	20.0

**Table 5:** Comparison of exponential decay time $\tau_{d}$ within 10s between solutes with different Stokes radius and diffusivity. The ‘different diffusivity $D_{s}$’ means that for Rs=3nm, Rs=5nm and Rs=9nm, the solute diffusivity is $1\times 10^{-10}$, $1\times 10^{-11}$, and $1\times 10^{-12}\;m^{2}/s$, respectively.

Molecule Size Rs (nm)	3	5	9
$\tau_{D}$ (h) with different $D_{s}$	1.4	8.9	48.8
$\tau_{D}$ (h) with fixed $D_{s}=1\times 10^{-11}$	8.4	8.9	10.3

**Figure A1:**
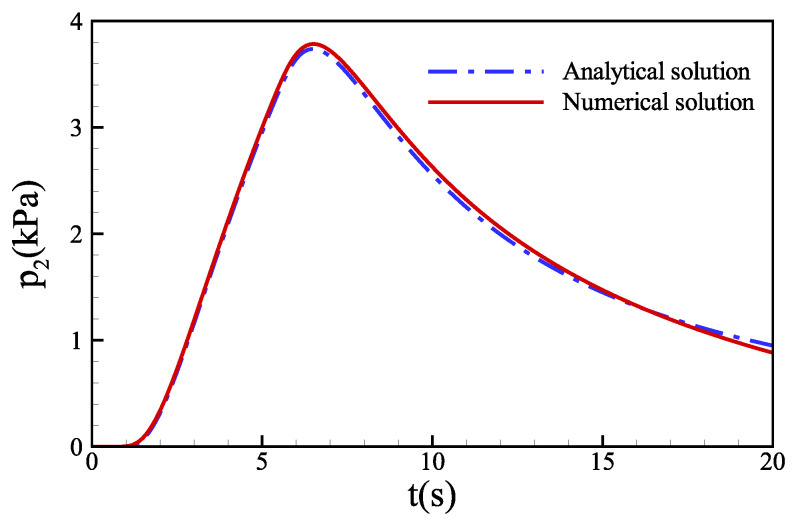
Comparison of numerical results with the analytical solution of pressure evolution at point $P_{2}$ for the proposed poroelasticity model.

**Figure A2:**
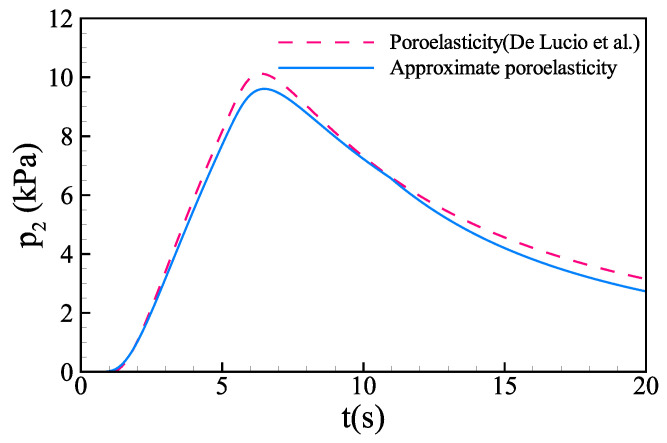
Numerical simulation results for pressure using approximate poroelasticity compared to results using full poroelasticity using an identical injection model and parameters [14].

**Figure A3:**
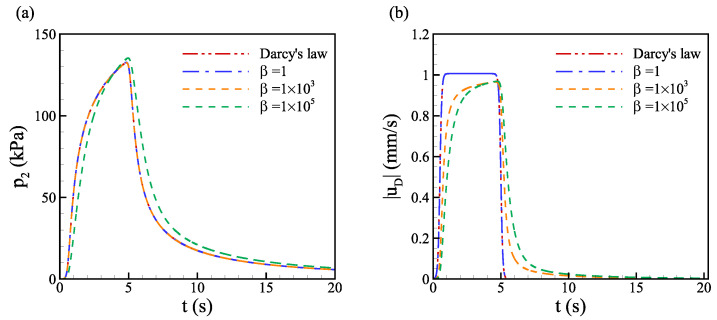
Effect of viscous effect with various viscosity ratios β on the evolution of (**a**) interstitial fluid pressure $p_{2}$ and (**b**) Magnitude of Darcy velocity $|u_{D}|$ at point $P_{2}$, 10 mm below the injection point.

**Figure A4:**
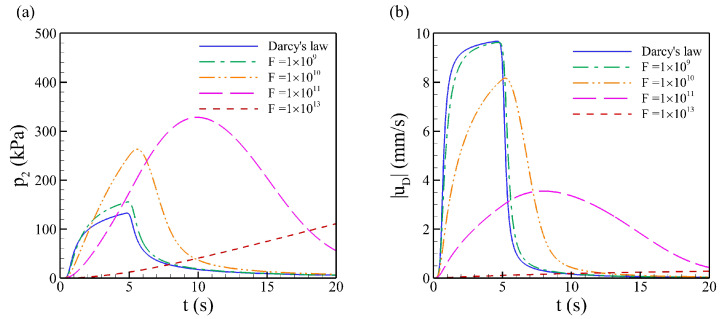
Effect of inertial forces with various Forchheimer coefficient *F* on the evolution of (**a**) interstitial fluid pressure $p_{2}$ and (**b**) Magnitude of Darcy velocity $|u_{D}|$ at point $P_{2}$, 10 mm below the injection point.

## Data Availability

The data that have been used are confidential.

## Appendix A

### Appendix A.1. Boundary Condition

The governing equations are solved through the open-source platform, OpenFOAM. The boundary conditions for the domain and the mesh convergence study for the multi-layered poroelastic domain are discussed in this section.

For the boundary condition, the left side and front side of the domain are symmetric planes. The B.C. on these two faces are symmetric boundary conditions for all fields, concentration *C*, velocity *u*, and pressure *p*. For the pressure field, the other two side faces are set to be the far-field boundary condition, i.e., a Dirichlet boundary condition $p=p_{\infty }$; on the top and bottom faces, zero gradients ∇p=0 are used to represent the upper epidermis layer, and the bottom bone layer is impermeable. For the velocity field, the far-field boundary condition (i.e., the zero gradient boundary) is used. The top and bottom faces of the domain are no-slip boundary u=0. For the concentration field, the faces are all zero gradient boundary conditions except the symmetric faces.

As for the discretization scheme, first, the Euler scheme is applied for the time derivative, and the ‘Gauss linear’ scheme is applied for the gradient of all fields. For the divergence term, the ‘bounded Gauss upwind’ is applied for the convection term in the momentum equation, while the ‘Gauss limited Vanleer’ scheme is applied for the convection term in the transport equation.

**Table A1:** Numerical schemes for the solver.

Terms	d(ϵC)/dt	∇C	∇·(uC)	$\nabla^{2}p$
Scheme	Crank-Nicolson 0.9	Gauss linear	Gauss limited Vanleer	Gauss linear corrected

The boundary of the explicit vessel wall on the side of the interstitial space is denoted as $\partial \Omega_{B}$ for blood vessels and $\partial \Omega_{L}$ for the lymphatic vessels. The boundary of the domain is denoted as ∂Ω. Variable n is the outside unit normal vector of the boundary surface, pointing to the interstitial space. First, we list the boundary conditions (B.C.s) on the lymphatic vessels. The pressure on the lymphatic vessel wall can be calculated by the fluid flux determined by Starling’s law, as shown in Equation (A1) [68]:

(A1) $$\begin{array}{cc} \; & \;-\frac{k}{\mu }{\nabla p|}_{\partial \Omega_{L}}=\delta_{valve}L_{pl}\varepsilon \left(p_{i}-p_{l}\right)n. \end{array}$$

The boundary conditions for the concentration on the lymphatic walls are determined by the improved Kedem–Katchalsky model, i.e., $J_{ls}{|}_{\partial \Omega_{L}}=J_{l}\left(1-\sigma \right)\left(C_{l}+C\right)/2+P_{l}^{\alpha }\left(C-C_{l}\right)$. The fluid flux is determined by Starling’s law, i.e., Equation (A1). The boundary condition is shown in Equation (A2) (a), which represents that the diffusion and convection of drug molecules through the vessel wall being equal to the solute flux through the vessel wall, which is governed by the proposed improved K-K model in the methodology section:

(A2) $$\begin{array}{cc} {-D\varepsilon \nabla C|}_{\partial \Omega_{L}}-\frac{k}{\mu }\nabla p{{|}_{\partial \Omega_{L}}C|}_{\partial \Omega_{L}} & =J_{ls}{|}_{\partial \Omega_{L}},\\ {\;\nabla C|}_{\partial \Omega_{L}}=(\frac{k}{\mu }{\nabla p|}_{\partial \Omega_{L}}{C|}_{\partial \Omega_{L}}-J_{ls}{|}_{\partial \Omega_{L}})/\left(D\varepsilon \right) & =\left(J_{l}C-J_{ls}\right)/\left(D\varepsilon \right), \end{array}$$

The boundary conditions on the blood vessel walls are listed below. The fluid velocity on the blood vessel wall is modeled by Starling’s law:

(A3) $$u_{b}{|}_{\partial \Omega_{B}}=-L_{pb}\varepsilon \left(p_{v}-p_{l}-\sigma \left(\pi_{v}-\pi_{i}\right)\right)n,$$

similar to the boundary condition for pressure on the lymphatic vessel, the pressure on the blood vessel is as follows:

(A4) $$\;-\frac{k}{\mu }{\nabla p|}_{\partial \Omega_{B}}=L_{pb}\varepsilon \left(p_{v}-p_{i}-\sigma \left(\pi_{v}-\pi_{i}\right)\right)n.$$

To address the fluid balance after the injection in the long term, we assume $J_{b}=J_{l}$, which gives a non-zero resting interstitial pressure $p_{\infty }$. The equivalence of lymphatic drainage rate and blood perfusion rate is shown in Equation (A5),

(A5) $$L_{pb}\frac{S_{b}}{V}\left[p_{v}-p_{\infty }-\sigma \left(\pi_{v}-\pi_{i}\right)\right]=L_{pl}\frac{S_{l}}{V}\left(p_{\infty }-p_{l}\right),$$

substituting the values for all parameters and solving the above fluid balance equation, we can obtain a non-zero interstitial pressure at equilibrium $p_{\infty }$. For our baseline case, $p_{\infty }=82.4\;\mathrm{Pa}$.

### Appendix A.2. Injection Model

Instead of resolving the needle geometry where the drug is injected, a spatial and temporal variable spherical injection profile is adopted, which represents the spherical fluid bolus [11,13,14]. The injection model with spatial variable injection rate is achieved by the hyperbolic tangent function of distance from the injection point, as shown in (see Equation (A6)). For the injection rate, the smoothed injection rate profile based on hyperbolic tangent functions is adopted here Equation (A7) to help alleviate the sharp change of mass and enhance the convergence of the numerical algorithm.

(A6) $$q_{s}=\frac{1}{2}\left(1-\tanh\left(\gamma \left(d\left(x\right)-R_{i}\right)\right)\right),$$

where the volumetric injection rate $q_{0}$, time duration $t_{i}$, and total injection volume $V_{i}$ need to be specified. The volumetric injection rate $q_{0}=V_{i}/t_{i}/\left(\frac{4}{3}\pi {R_{i}}^{3}\right)$, where $R_{i}$ is the radius of the injection ‘sphere’. A previous study [69] showed that longer injection time and larger needle radius help to reduce the injection force and ease patients’ pain. For the injection model described by Equation (A6), the sphere represents the fluid plume coming out of the needle, and the radius $R_{i}$ could vary with the needle, which ranges from 0.08–3.8 mm in inner diameter. Here, we take $R_{i}=1$ mm as the baseline value for the sphere plume.

(A7) $$q_{t}\left(t\right)=A(\tanh\left(B\left(t-t_{s}\right)\right)-\tanh\left(B\left(t-t_{e}\right)\right)$$

where $t_{s}=0.5$ and $t_{e}=5.0$ is the start and end time for the injection. A and B are adjustable constants, here, we take A=5/9, B=7, the same as in [14].

### Appendix A.3. Qualification of the Poroelasticity Model

Three observation points on the axis of symmetry are chosen to study the evolution of pressure, $P_{0}$, $P_{1}$ and $P_{2}$, where $P_{0}$ is the injection point, and $P_{1}$ and $P_{2}$ are 2 mm and 10 mm below the injection point, respectively. To verify the validity and accuracy of the proposed approximate poroelasticity model, the results for pressure evolution are quantitatively compared to the analytical solution and numerical results of the full poroelasticity model [14].

#### Appendix A.3.1. Comparison against the Analytical Solution

An analytical solution of Equation (5) is obtained in an infinite domain, along with a homogeneous initial condition, i.e., p(x,t=0)=0, using the Fourier transform on space and Laplace transform on time sequentially. For a clear presentation, the hydraulic conductivity K=k/μ is used and injection source term $q_{r}$ is modeled as $q_{d_{s}}H_{h}\left(t_{i}-t\right)\delta_{h}\left(x-x_{i}\right)$, where $q_{d_{s}}$ represents the injection volume, and $H_{h}$ and $\delta_{h}$ are approximations of the Heaviside function and delta function, respectively. Here, the Fourier transform of a variable *f* is denoted by $\tilde{f}\left(\xi \right)=F\left[f\left(x\right)\right]$, where ξ is the independent variable in the Fourier space, and its Laplace transform is denoted by $\hat{f}\left(s\right)=L\left[f\left(t\right)\right]$, where *s* is the independent variable in the Laplace space. After applying the Fourier transform to Equation (5), and its initial condition, and then the Laplace transform, the following algebraic equation for $\hat{\tilde{p}}$ is obtained

(A8) $$s\hat{\tilde{p}}-\tilde{p}\left(\xi ,t=0\right)=-D_{e}K\xi \cdot \xi \hat{\tilde{p}}+D_{e}q_{d_{s}}{\tilde{\delta }}_{h}\left(\xi \right){\hat{H}}_{h}\left(s\right),\;\tilde{p}\left(\xi ,t=0\right)=0.$$

Immediately, $\hat{\tilde{p}}$ is solved from the above equation, namely:

(A9) $$\hat{\tilde{p}}\left(\xi ,s\right)=D_{e}q_{d_{s}}{\tilde{\delta }}_{h}\left(\xi \right)\frac{\hat{H}\left(s\right)}{s+D_{e}K\xi \cdot \xi }=D_{e}q_{d_{s}}{\tilde{\delta }}_{h}\left(\xi \right)L\left[H_{h}\left(t_{i}-t\right)\right]L\left[e^{-D_{e}K\xi \cdot \xi t}\right].$$

The Fourier transform of *p*, i.e., $\tilde{p}$, is obtained by performing the inverse Laplace transform to $\hat{\tilde{p}}$. Noticing that $\hat{\tilde{p}}$ is a product of the Laplace transforms of two functions, the convolution theorem is applied and $\tilde{p}$ is

(A10) $$\begin{array}{c} \tilde{p}\left(\xi ,t\right)=D_{e}q_{d_{s}}{\tilde{\delta }}_{h}\left(\xi \right)H_{h}\left(t_{i}-t\right)*e^{-D_{e}K\xi \cdot \xi t}=D_{e}q_{d_{s}}{\tilde{\delta }}_{h}\left(\xi \right)\int_{0}^{t}H_{h}\left(t_{i}-\tau \right)e^{-D_{e}K\xi \cdot \xi \left(t-\tau \right)}d\tau \\ =D_{e}q_{d_{s}}\int_{0}^{t}H_{h}\left(t_{i}-\tau \right)F\left[\delta_{h}\left(x-x_{i}\right)\right]F\left[\frac{1}{{\left(4\pi D_{e}K\left(t-\tau \right)\right)}^{d_{s}/2}}e^{-\frac{x\cdot x}{4D_{e}K\left(t-\tau \right)}}\right]d\tau . \end{array}$$

Similarly, $\tilde{p}$ is also a product of the Fourier transforms of two functions. After applying the convolution theorem again, the solution of Equation (5) is obtained, namely:

(A11) $$\begin{array}{c} p\left(x,t\right)=D_{e}q_{d_{s}}\int_{0}^{t}\frac{H_{h}\left(t_{i}-\tau \right)}{{\left(4\pi D_{e}K\left(t-\tau \right)\right)}^{d_{s}/2}}\delta_{h}\left(x-x_{i}\right)*e^{-\frac{x\cdot x}{4D_{e}K\left(t-\tau \right)}}d\tau \\ =D_{e}q_{d_{s}}\int_{0}^{t}\frac{H_{h}\left(t_{i}-\tau \right)}{{\left(4\pi D_{e}K\left(t-\tau \right)\right)}^{d_{s}/2}}\int_{-\infty }^{\infty }\delta_{h}\left(\chi -x_{i}\right)e^{-\frac{\left(x-\chi )\cdot (x-\chi \right)}{4D_{e}K\left(t-\tau \right)}}d\chi d\tau . \end{array}$$

Since integrals in the analytical solution Equation (A11) can rarely be computed exactly, we have to evaluate them numerically, given $H_{h}\left(t_{i}-t\right)$ and $\delta_{h}\left(x-x_{i}\right)$. The spatial integral is performed in a spherical coordinate, namely:

(A12) $$\begin{array}{c} f\left(x,t,\tau \right)=\int_{-\infty }^{\infty }\delta_{h}\left(\chi -x_{i}\right)e^{-\frac{\left(x-\chi )\cdot (x-\chi \right)}{4D_{e}K\left(t-\tau \right)}}d\chi \\ =\int_{0}^{2\pi }d\phi \int_{0}^{\pi }d\theta \int_{0}^{R_{I}}\delta_{h}\left(\chi -x_{i}\right)e^{-\frac{\left(x-\chi )\cdot (x-\chi \right)}{4D_{e}K\left(t-\tau \right)}}r^{2}\sin\left(\theta \right)dr,\\ \chi =x_{i}+\left\{r\sin\left(\theta \right)\cos\left(\phi \right),r\sin\left(\theta \right)\sin\left(\phi \right),r\cos\left(\theta \right)\right\}, \end{array}$$

with a given τ, *t*, x, and $x_{i}$, and the Gaussian quadrature rule is applied. Here, $R_{I}$ should be infinite theoretically but is finite in practice, because the smoothed delta function $\delta_{h}\left(x\right)$ is non-zero only in a small region around x. Then, Equation (A11) is rewritten as:

(A13) $$p\left(x,t\right)=D_{e}q_{d_{s}}\int_{0}^{t}g\left(x,t,\tau \right)d\tau ,$$

where

(A14) $$g\left(x,t,\tau \right)=\frac{H_{h}\left(t_{i}-\tau \right)}{{\left(4\pi D_{e}K\left(t-\tau \right)\right)}^{d_{s}/2}}f\left(x,t,\tau \right).$$

To compute the integral in Equation (A13), the compound mid-point rule is employed, namely,

(A15) $$\int_{0}^{t}g\left(\tau ,t\right)d\tau =\sum_{n_{t}=1}^{N_{t}}\int_{(n_{t}-1)\Delta t}^{n_{t}\Delta t}g\left(\tau ,t\right)d\tau \approx \sum_{n_{t}=1}^{N_{t}}g\left(\tau =\frac{2n_{t}-1}{2}\Delta t,t\right)\Delta t,$$

with *t* given, where Δt is the time step and $N_{t}=t/\Delta t$ is the number of partitioned time intervals.

In the present study, we use $H_{h}\left(t_{i}-t\right)=q^{t}\left(t\right)$ and $\delta_{h}\left(x-x_{i}\right)=q^{s}\left(x\right)$, which are detailed in Appendix A.2, although the analytical solution Equation (A11) works for any other smoothed or even the exact Heaviside and delta functions. The reported results used 10 Gaussian quadrature points along each coordinate and Δt was fixed to be 0.05, and we have checked that almost the same results were obtained using half the number of the Gaussian quadrature points and doubled the time step Δt. Using the same boundary condition as the analytical solution, we obtain the evolution of the pressure for the proposed poroelasticity model at the point $P_{2}$. The pressure evolution shows good agreement with the analytical solution, as shown in Figure A1. To further verify the effectiveness and accuracy of the proposed model, we also quantitatively compare the results of pressure evolution against the previously obtained poroelasticity model.

#### Appendix A.3.2. Comparison against the Full Poroelasticity Model

To further validate the accuracy of the approximate poroelasticity model, we quantitatively compare our results to [14], where the full poroelasticity model was implemented. We use the same injection model, a sphere bolus with radius R=0.1mm. Here, the domain size and injection volume are 80×80×100mm and 2 mL, respectively. Elasticity and permeability are E=80 kPa, $E_{v}=267\;\mathrm{kPa}$ and $k=1\times 10^{-13}$ m${}^{2}$, respectively. As shown in Figure A2, the time evolution of pressure at point $P_{2}$, 10 mm below the injection point, using the approximate poroelasticity model, agrees well with the results using the full poroelasticity model. The small discrepancy could also come from the numerical method. The finite element method is used in [14], while in our simulation, the discretization is based on the finite volume method.

### Appendix A.4. Non-Darcy Flow Effect

By implementing the Darcy–Brinkman–Forchheimer equation, the non-Darcy flow effect is investigated on the pressure build-up. Darcy’s law is simple and widely used to simulate fluid flow across the continuous porous medium. However, it cannot take into account the boundary effect and the inertia effect [70]. The extracellular matrix is full of fibrous proteins, such as collagen and elastin. Significant departures from Darcy’s law first occur at Reynolds numbers on the order of *O* (1) [71] in the fibrous porous medium. The effect of boundary is important for two layers of fluid-porous medium [72] and flow across a microvessel wall [20].

The axial pressure gradient can be represented as the sum of two terms, one is linear in the velocity (viscous contribution), and the second quadratic in the velocity (inertia contribution) [70,73]. The inertia effect is large during the injection, as the pressure and fluid velocity are large. Thus, the inertia drag may not be neglected in such a condition. Here, the Darcy–Brinkman–Forchheimer equation is implemented to investigate the role of viscous and inertial effects on pressure increase and relaxation. Darcy’s law is typically used to approximate fluid flow in the porous medium with a small velocity or Reynolds number.

The Brinkman equation is used to replace Darcy’s law to take into account the effect of the boundary, the top surface of the domain, and the porous vessel wall. During the injection the velocity of fluids is rather large, i.e., *O* (1m/s), and the Darcy velocity reaches O(10mm/s) at the end of the injection. The Reynolds’ number, $Re=\frac{\rho UR}{\mu }$, can reach to *O* (10). The advantages and applicable tissue types of the Darcy model, the Darcy–Brinkman model, and the Darcy–Brinkman–Forchheimer model are listed in the table below [70].

**Table A2:** Comparion of Darcy’s law, Brinkman model, and Forchheimer model.

Model	Feature	Applicable Tissues
Darcy model	Darcy damping	Tumor tissue; perfused muscle tissue
Brinkman model	Boundary effect and viscous effect	Microvessel walls; fluid-porous interface
Forchheimer model	Inertial effect and drag force	Tissue enduring processes with large inertial effects

Here, we take into account both the inertial and viscous loss. The general equation governing fluid flow, i.e., the Darcy–Brinkman–Forchheimer equation, reads:

(A16) $$-\nabla p=\frac{\mu }{k}u_{D}-\frac{\mu_{e}}{\varepsilon }\nabla^{2}u_{D}+\frac{\rho \varepsilon F}{2}\left|u_{D}\right|u_{D}$$

where, the magnitude of velocity $|u_{D}|=\sqrt{u_{D}\cdot u_{D}}$, *F* is the Forchheimer coefficient, and $\mu_{e}$ is the effective viscosity. Balancing the Darcy damping term $\frac{\mu }{k}u_{D}$ and the Brinkman viscous term $\frac{\mu_{e}}{\varepsilon }\nabla^{2}u_{D}$, we can obtain the length scale $L_{b}=\sqrt{k/\varepsilon }$. Over this length scale, the viscous effect is important. The darcy number, defined as $Da=k/L^{2}$, is widely used to quantify the ratio of pore scale and the characteristic length scale [72]. Setting the ratio of the effective viscosity and the fluid viscosity as β, $\beta =\mu_{e}/\mu$, β/εDa is the ratio of the Darcy damping term and the Brinkman viscous term. Taking the characteristic length scale $L=L_{b}$ and β=1, we have β/εDa=1, which indicates the viscous force balances out the damping force of the porous medium. Taking the thickness of the skin layer *O* (1mm) as the characteristic length scale, $\beta /\varepsilon Da=\beta 10^{-5}$. For the scaling analysis, the viscous effect over the large length scale is important when the effective viscosity ratio reaches $10^{5}$. Thus, we vary the viscosity ratio β from 1 to $10^{5}$. The evolution of pressure and velocity for different viscosity ratios β is shown in Figure A3. The numerical results are consistent with the above theoretical analysis. The effect of viscosity on pressure is negligible until the viscosity ratio β increases to $10^{5}$. However, the difference is still small for both pressure and velocity at such a large viscosity ratio.

Comparing the Darcy damping term and Forchheimer inertial term, we define the Forchheimer number as $F_{r}=\rho \varepsilon Fk/2\mu$. When $u_{D}$ is larger than or of the same order of magnitude as $1/F_{r}$ (i.e., $u_{D}∼F_{r}\left|u_{D}\right|u_{D}$), the Forchheimer term is as important as the Darcy damping, and the inertial effect is not negligible. Varying the Forchheimer coefficient *F* from $10^{9}$ to $10^{13}$, the Forchheimer number ranges from 1 to $10^{4}$. The pressure at point 2 mm below the injection point $p_{2}$ varies non-monotonically with the Forchheimer number. Pressure $p_{2}$ first increases with *F* as the inertial drag increases and the fluid accumulates near the injection point. As *F* increases to $10^{13}$, the pressure profile changes as the fluid can hardly spread to the observation point. The Forchheimer coefficient *F* is closely related to the Reynolds number. For the injection in the subcutaneous layer with maximum Reynolds number Re = 1–10, the magnitude of F is $10^{4}$, which means the inertial effect is negligible.
